# Correspondence

**Published:** 1859-08

**Authors:** 


### Correspondence.

### WARPING OF PLATES.

I know very well that this subject of "Warping of Plates" has been written about a great deal; still I think it is not exhausted yet. The following method, which is very simple, and easily tried, I find to work quite satisfactorily. After the teeth are arranged upon the plate, and fastened with wax in the usual way, I put on my investment in the following manner. Lay the case on a piece of paper, with the coronal ends of the teeth downward. Then take a piece of No. 15 wire, bend it so as to fit closely to the teeth, (almost touching them,) letting it extend back as far as the backside of the plate on each side: lay it on the paper, and then put on your investment, building it up around the teeth until it forms a rim as high as any portion of the plate; not letting it cover but very little plate, just enough to hold it in its place. Place another wire, like the first, near the base of the teeth. After the investment has hardened, turn it over and fasten the case on a piece of charcoal or pumice stone, with binding wire, and then fill up under the plate with asbestos. Heat up with a large flaring blaze of the blow-pipe. You can heat up in a few minutes, without much danger of warping the plate, or cracking the teeth. The smaller the quantity of investment around the teeth, the better; sufficient to hold the teeth *in situ* is all that is necessary. I have not had a plate to warp so as to trouble me since I adopted this method.

As soon as the case is soldered, it should be placed on a board, or some charcoal, with the coronal ends of the teeth downward, so as to cool slowly and equally. I will let others explain the theory—I will simply state the fact.

Yours, truly, H. M. SHEERAR.

Wellsville, N. Y., July 12, 1859.

---

MESSRS. EDITORS:—I have read with much pleasure your first monthly issue—the July number of the *Register*—and desire to say that if you can fill each subsequent number with as entertaining and as useful matter as this contains, you will have accomplished more than I have been disposed to think was possible. I have entertained the opinion that a monthly dental periodical, of respectable size, could not be sustained, and chiefly because of a want of sufficient suitable matter; not but what there is an abundance of material—for no profession has made such rapid strides, and accomplished so much in a given time, as the dental profession. And where there are such advances, there must of necessity be much to communicate—but the materal remains unwrought, and therefore unavailable, which is all owing to that almost insurmountable obstacle, *indisposition* on the part of the great mass of the profession to put their thoughts, modes of practice, and experience, on paper. Therefore they do not contribute, and therefore the lack of matter. But, perhaps, the demand made upon them by the issue of your monthly—and the one recently announced in this city—may induce an effort, on the part of the profession generally, to sustain the enterprise by

> Those writing who never wrote before,
> And those who did, to write the more.

For the credit of the profession, I hope it may be so.

As the time approaches, there is some talk about the next meeting of the American Dental Convention. So far as I have been able to ascertain, there will be some twenty or more delegates (not counting the ladies,) from this city, many of whom will probably advocate the formation of a "National Society on a Representative basis."

I regret to see the opposition manifested by some of your contributors, as well as by a number of the profession in New York, to this project. They are, I think, premature in their opposition. I would have all such wait until it properly comes up for consideration, when it may assume a different and more pleasing phase than it now presents to them. One special, and I conceive important result, of such an organization, would be the formation of State and city associations, for it being established on a representative basis, would render necessary such associations to secure a representation, and this result alone, independent of its other advantages, should commend it, I think, to the support of the profession generally, for State and city associations, with their frequent meetings, are more productive of good, professionally and socially, than conventions or national societies ever can be. Still, these larger convocations have their office—they enlarge the intercourse and liberalize the mind, and thus increase the usefulness of those participating in them. Therefore I think that there cannot be too many of them.

A few days since, while in the neighborhood, I paid a visit to the grave of Elisha Townsend; and as I leaned upon the railing, a flood of recollections passed through my mind, running back to our first acquaintance, many years ago. How well I remember his desire to bring about a more kindly feeling among the dentists of this city at that day, and his willingness to impart whatever might advantage his professional brethren; then his subsequent public efforts and labors for the same object, with which all are familiar; but particularly did I remember and dwell upon his sociable, companionable qualities, as well as his professional excellence. Surely the usefulness of such a professional life as his cannot well be over-estimated. Allow me to give you one from many illustrations that might be mentioned. A dentist from a neighboring town, in no practice, called upon him to have a tooth filled. He did not disclose his profession, and with mirror in hand noticed the operation from beginning to end. On his return home, with the first patient that presented himself, he put in practice the method used in plugging his own tooth, and after five distinct efforts upon the cavity, he succeeded in filling it to his satisfaction. He remarked afterwards, when narrating the occurrence, that he dreamed of it that night, his mind being so engrossed, and that by repeated subsequent efforts, he was enabled to fill a tooth in a manner he never supposed possible, and that he had learned more in plugging teeth, from seeing his own tooth filled, than he had learned during his whole previous practice.

Dr. Flagg the elder has gone to Paris, chiefly for the benefit of his sight, which has become somewhat impaired. Professionally, there is nothing new. A large representation is looked for at Niagara. All the profession and their wives are likely to be there.

If agreeable to you, I may drop you an occasional letter.

Yours, O. U. C.

**Philadelphia, July 13, 1859.**

"Agreeable?" Most assuredly. Yes; desirable.

